# Cardiometabolic Indices after Weight Loss with Calcium or Dairy Foods: Secondary Analyses from a Randomized Trial with Overweight/Obese Postmenopausal Women

**DOI:** 10.3390/nu14051082

**Published:** 2022-03-04

**Authors:** Jasminka Z. Ilich, Pei-Yang Liu, Hyehyung Shin, Youjin Kim, Yichih Chi

**Affiliations:** 1 Institute for Successful Longevity, Florida State University, Tallahassee, FL 32306, USA; 2 School of Exercise and Nutrition Sciences, University of Akron, Akron, OH 44325, USA; liu4@uakron.edu; 3 Department of Social Welfare, Pusan National University, Pusan 46241, Korea; hshin@pusan.ac.kr; 4 GNC Holdings, LLC, Pittsburg, PA 15222, USA; youjinkim@gmail.com; 5 Independent Researcher, Fremont, CA 94539, USA; yichih.chi@gmail.com

**Keywords:** calcium and vitamin D supplements, low-fat dairy foods, cardiometabolic risk factors, weight loss, blood pressure, serum apolipoproteins, adipokines, serum vitamin D

## Abstract

The role of dairy foods and calcium/vitamin D supplements in cardiometabolic diseases is unknown. The objective of this secondary analysis is to investigate cardiometabolic risk factors changes after a 6-month weight-loss intervention in overweight/obese postmenopausal women divided in three groups: Ca+vitamin D supplements (S); low-fat dairy foods (D; 4–5 servings/day); or control/placebo pills (C), as complements to hypocaloric diets. The original study focused on bone/body composition. This analysis included blood pressure (BP), and serum triglycerides, lipids (including apoproteins Apo1 and ApoB), adipokines, and C-reactive protein in *n* = 97 participants who finished with complete data points. Systolic BP decreased 5.1%, 4.8%, and 1.8% in S, D, and C groups, respectively (*p* < 0.05 for S and D vs. baseline and vs. C at 6 months). Reduction in triglycerides and ratio of total cholesterol (TC)/high-density lipoproteins cholesterol (HDL-C) was the highest in S, while the reduction in TC and LDL-C was the highest in D group (all *p* < 0.05). Leptin and ApoB significantly decreased and adiponectin and ApoA1 increased in all groups. In conclusion, although the C group’s participants experienced an improvement in some of the cardiometabolic indices with weight loss, those in the S and D groups showed significantly better results in most of the outcomes, indicating the beneficial effects of low-fat dairy foods and/or Ca+vitamin D intake as complements to a hypocaloric diet.

## 1. Introduction

According to the 2000–2019 global assessment, cardiometabolic diseases, including cardiovascular diseases (CVDs), stroke and type 2 diabetes, remain the major causes of mortality and morbidity in the United States and throughout the world [1]. Postmenopausal women, particularly those who are overweight/obese, are at elevated risk due to a combination of factors, most notably the cessation of estrogen and the excess of body fat with its pro-inflammatory secretory products. Aging itself leads to physiological changes among which low-grade chronic inflammation is a driver for many chronic diseases, including cardiometabolic [2,3]. In addition to overweight/obesity and the comorbidities associated with it, some biomarkers even in normal-weight individuals, including postmenopausal women, have been identified as risks for cardiometabolic diseases [1]. These include high blood pressure, aberrant serum lipids (high cholesterol, triglycerides, and atherogenic lipoproteins) and elevated serum glucose and pro-inflammatory serum markers, such as adipokines (e.g., leptin) and C-reactive protein (CRP).

Dairy foods and milk are nutrient-dense foods containing many essential nutrients, including calcium, potassium, and vitamin D, all three of a public health concern [4] because of their overall low intake. Additionally, dairy foods are rich in high-quality protein, some beneficial fatty acids, as well as multiple other minerals and vitamins, including magnesium, selenium, zinc, and vitamins A, B_12_, pantothenic acid, and riboflavin. Fermented dairy foods (yogurts and cheeses) also provide probiotics to the diet. Dairy foods and some of the individual nutrients in them (most notably calcium) have been implicated in cardiometabolic risks modulation, although with inconsistent findings and not always clear health benefits [5]. There have been many suggested reasons for the inconsistent findings (outside of the scope of this paper), but one notable reason is that, in many studies, there was no clear distinction between fermented and non-fermented amounts/proportions consumed within the dairy food matrix [6,7]. It was reported that fermented dairy foods were associated with lower insulin resistance and oxidative stress, whereas non-fermented (primarily milk) were associated with higher blood pressure, lower high-density lipoproteins cholesterol (HDL-C) [6] and inflammation [8], the latter possibly due to galactose (pro-inflammatory monosaccharide) in milk [9]. Therefore, it is possible that dairy foods have a dual effect, depending on the kind and amount consumed, and may be acting at multiple platforms, affecting not just serum lipids and blood pressure, but also inflammation and oxidative processes, all implicated in cardiometabolic risks.

The role of calcium (Ca) itself in body weight, body adiposity [10] and even in fracture risk [11] is unsettled as well. Its role in cardiometabolic diseases and their multiple risk factors is even more ambiguous. A study reanalyzing the Women’s Health Initiative data with the metanalysis of other published studies [12] reported the increased risk of myocardial events with calcium supplements with and/or without vitamin D, mostly due to increased vascular calcification. While vascular calcification is still being debated within the bone loss issues [13], its role in CVDs was subsequently refuted multiple times, including in a study based on the results from the 5-year clinical trial [14], or from meta-analyses [15], or from the Korean National Cohort study, the latter one recommending that vitamin D be added to Ca supplements [16]. To add to the controversy, some studies reported the worsening of the serum lipid profile with higher serum Ca [17], while others showed the opposite effect with Ca supplementation [18]. Despite the controversies, the majority of findings reveal no adverse effect of Ca supplements on CVDs risks, especially when taken with vitamin D. Additionally, the multiple beneficial effects of either Ca [19] or dairy foods [20] on cardiometabolic risks have been reported, including those on blood pressure [21]. In view of the above, it is important to investigate these relationships more closely in the randomized clinical trials, as presented in this study.

Recently, we published the results from a 6-month weight loss clinical trial in overweight/obese postmenopausal women. All participants consumed hypocaloric diets complemented with either calcium + vitamin D supplements, low-fat dairy foods, or placebo pills (control) [22]. The weight loss was moderate, but the beneficial effects in lean and fat tissues were the highest in participants on low-fat dairy foods. The bone health outcomes were the best in the participants receiving calcium + vitamin D supplements, compared to those in the control group.

These results prompted us to examine further the data from this weight-loss study and investigate the cardiometabolic risk factors outcomes during a 6-month intervention. Specifically, in this study, we examine the change in blood pressure, serum glucose, insulin, lipids (including apoproteins A and B), adipokines (leptin, adiponectin), and pro-inflammatory marker, CRP. We hypothesize that the hypocaloric diets with either Ca + vitamin D supplements or increased low-fat dairy foods will be associated with greater benefits in cardiometabolic factors compared to the control group (only hypocaloric diet).

## 2. Methods

### 2.1. Weight Reduction Protocol and Dietary Plan

The weight reduction protocols, described in detail recently [22], involved moderate energy restriction (~85% of energy needs) for the participants randomly divided in three groups: (1) Dairy group (D; including 4–5 servings/day of low-fat dairy foods); (2) Supplement group (S; with prescribed pills, each containing 315 mg Ca + 200 IU of vitamin D/day); and (3) Control group (C; with placebo pills). The S and C codes were double blinded and the allocator at Bayer, HealthCare LLC (pill manufacturer), concealed the codes until the end of the study. The energy intake for dietary plan was designed using the Mifflin St. Jeor equation [23] to estimate each participant’s energy requirement for the weight maintenance, from which ~500 kcal was subtracted to elicit a weight loss of 0.5–1 kg/week. The behavioral modification practices for all participants (control and intervention) were regularly conducted, as previously described [22,24].

Both food frequency questionnaire and 3-day dietary records were collected upon the entry into the study to estimate initial energy and Ca/vitamin D intake. Participants in the D group were instructed to consume 4–5 servings of low- or reduced-fat dairy foods (e.g., cheese, skim or 1% fat milk, yogurt, pudding, low-fat ice cream and frozen yogurt), to reach the intake of ~1500 mg Ca/day and ~600 IU/day vitamin D total, including other usual food items. Participants in the S group received Ca citrate + vitamin D tablets (adjusted individually to provide the total of ~1500 mg Ca/day and ~600 IU/day of vitamin D) and the C group received placebo pills provided by Bayer, HealthCare LLC, Morristown, NJ. The compliance with Ca supplements/placebo was monitored by pill count, while that of dairy food intake by dietary records. In all cases, 24 h urine (see below) was analyzed for Ca/creatinine levels for overall kidney functioning to double check the compliance [22].

During the first 3 months of the “run-in” period, all participants attended the biweekly 2 h group sessions (with 5–8 members of the same intervention group together), which incorporated the multimodal cognitive-behavioral intervention, as well as nutritional education [24,25]. Subsequently, these sessions were reduced to one monthly meetings until the end of the study.

### 2.2. Participants

The selected participants (*n* = 97 in this analysis) were postmenopausal overweight/obese women, generally healthy, able to live independently and free of chronic diseases, as described previously [22,24]. They could not be taking cholesterol-lowering medications or others known to affect bone metabolism (e.g., hormone replacement therapy or corticosteroids) or blood pressure for the past 3 months. They could not be currently smoking > 1 pack of cigarettes/day or consuming >2–3 drinks/day of alcohol or caffeine. They also could not be involved in excessive exercise (although moderate exercise was allowed and encouraged) in the past 3 months.

In addition, only participants with total dietary Ca intake of ≤800 mg Ca/day (as screened by the initial food frequency questionnaire) were enrolled. If the qualifying participant was taking Ca supplements, she was asked to stop and was allowed 1 month of equilibration before enrolling in the study. Other mineral and vitamin supplements were allowed, but were recorded and accounted for in all analyses. The study protocol was approved by the Institutional Review Board at the Florida State University and the signed consent was obtained from each participant.

### 2.3. Measurements

#### 2.3.1. Anthropometry, Body Composition and Clinical Chemistry

All measurements were performed at baseline and 6 months visit, as described previously in detail [22,24]. Briefly, weight and height were measured by standardized procedures in normal indoor clothing without shoes. Systolic and diastolic blood pressure (SBP and DBP, respectively) was recorded in duplicates in a sitting position after a 5–10 min. rest, using a digital device (Omron Healthcare, Inc., Bannockburn, IL, USA). The dual energy x-ray absorptiometry (iDXA, GE Medical Systems, Madison, WI, USA) was used for the bone and body composition (lean and fat tissues) assessment, according to the manufacturer’s specifications [22,24].

Blood was collected after an overnight fast. Serum was separated from red blood cells after centrifugation and analyzed for routine tests, including Ca, protein, and creatinine, as well as serum lipids by the contracting laboratory (Quest Diagnostics, San Capistrano, CA, USA). Remaining serum samples were stored at −80 °C and later analyzed using commercially available enzyme-linked immunosorbent assay kits for leptin and adiponectin (ALPCO Diagnostics, Salem, NH, USA) and for 25 hydroxy vitamin D (25(OH)D) and parathyroid hormone (PTH) (Immunodiagnostic Systems, Fountain Hills, AZ, USA). The reagents for each assay were prepared and steps performed according to the manufacturer’s instruction with coefficients of variations <10%. Each sample was tested in duplicate.

A 24 h urine sample (after each participant received written/and oral instructions), was collected, the urine volume was measured and the aliquoted samples were routinely analyzed for calcium, sodium, phosphorus, magnesium, and creatinine (Quest Diagnostics, San Capistrano, CA, USA). These tests served to check for the for overall health, as well as for the compliance with dairy/Ca/placebo pills diets [22,26].

#### 2.3.2. Dietary and Physical Activity Assessment

The 3-day dietary records (2 week and 1 weekend day) were collected upon entering the study and subsequently on a biweekly basis in the first 3-month run-in period and then at the end of the study (6 months). The Food Processor^®^ (ESHA Research, Salem, OR, USA) was used to analyze the records for energy and all nutrients. The intakes of vitamin and mineral supplements were carefully recorded and included in the total nutrient analysis, as described previously [22,24,27].

Physical activity was assessed using a modified Allied Dunbar National Fitness Survey [22,28,29], to assess participation in recreational and sport activities, as well as other everyday activities, such as: heavy housework, home improvement, gardening, and walking. Each activity was assessed for frequency and duration and expressed in hours/week based on the average of the previous 4 weeks. The activities were added up to calculate the total activity score in hours/week [28]. The records were obtained at the beginning and end of the study. During the 3-month run-in period, the activity journals were kept and evaluated on a biweekly basis (same as dietary records). Participants also documented their activities over a 24 h period (for 2 weeks and 1 weekend day), including the number of hours of sleep, utilizing the questionnaire by Bouchard et al. [29], from which energy expenditure as metabolic equivalents (MET) was calculated.

### 2.4. Statistical Analyses

Most of the exploratory analyses, including descriptives and the check for normality of variables, were performed via standard statistical methods using SPSS (version 25) statistical package (IBM^®^, Armonk, New York, NY, USA). The initial exploration also included correlational analyses between weight loss outcomes (e.g., BMI and body fat) and measured cardiometabolic risk factors (BP and serum markers) to obtain better focus for calculations regarding the objectives of the current study. The longitudinal data were analyzed by repeated outcome measures. For example, the repeated measures analysis of covariance (RM ANCOVA) was utilized to test a three factorial (Dairy (D) vs. Supplement (S) vs. Control (C) groups) design for continuous variables (anthropometries and clinical data); the focus was on change, both overall and as a function of the three groups, and their interaction with time. Other demographic covariates (age and education) were also examined/included. The difference among three treatment groups (inter) or difference between baseline and 6 months within the same group (intra) were computed as well. The level of significance was set at *p* ≤ 0.05.

For the original study that focused on the effects of weight loss on bone, muscle and adipose tissue, the power was calculated using the results from the Chao et al. study [30] reporting the effect of weight loss on bone mineral density. For this secondary analysis, the power was estimated from the randomized double-blind controlled trial of Ejtadeh et al. [31]. In that study, the consumption of yogurt (300 g/day) significantly improved serum lipids during the 6 week intervention. With at least 80% power to detect a significant (*p* < 0.05) difference and utilizing G*Power [32], it was determined that *n* = 30 per group would be large enough to detect desired differences.

## 3. Results

A total of *n* = 189 subjects participated in the baseline measurements with an attrition rate of about 30% after 6 months of follow-up. There were also missing data in some variables even among the subjects who completed the study, as reported earlier [22]. To avoid missing data handling in this secondary analysis, we included only participants without any missing data, or only those with full sets of all-data points. Therefore, the secondary analysis was performed on *n* = 97 participants, with *n* = 30, *n* = 37, and *n* = 30 for C, S, and D groups, respectively.

Table 1 presents baseline characteristics, body composition, serum analyzes, dietary intake of selected nutrients and physical activity of participants who finished the 6-month follow-up with complete data sets. No statistical difference was found in variables between continuing and noncontinuing participants. The anthropometric, blood pressure, body composition, dietary and physical activity variables showed fulfillment with inclusion criteria, as indicated earlier [22]. The serum parameters were within the reference range for insulin, triglycerides, HDL-C, TC/HDL-C ratio, adiponectin, 25(OH)D, and PTH. The values were elevated for glucose (slightly), TC, LDL-C, leptin, ApoB and hs-CRP, but were below the reference range for ApoA1 (Table 1).

Although the number of participants was reduced from *n* = 135 in the original study to *n* = 97 for this analysis, the relations among variables at baseline remained the same between those who finished the study and those who were excluded and similar as in the original study [22]. Table 2 presents the baseline results from the correlational analysis between body composition parameters (most affected by weight loss) and serum cardiometabolic indices. As expected, the strongest significant positive correlations were found between BMI and body fat with insulin, leptin and hs-CRP. The positive, although significantly weaker correlations were noted between BMI and SBP, DBP, glucose, triglycerides and ApoB and negative with HDL-C. Similar trends (except for blood pressure, triglycerides, and HDL-C) were noted regarding the weight itself. However, unlike BMI, but similar to body fat (both kg and %), the weight was significantly negatively related to 25(OH)D. Lean mass was significantly positively related to glucose, insulin, TC/HDL-C, leptin, ApoB, and hs-CRP, probably reflecting the overall body mass/size in this correlational analysis.

The weight loss and changes in body composition parameters from the original study have been presented in detail [22]. Briefly, overall weight loss was moderate, with the highest percentage decrease in all anthropometric variables and total body and android fat in the D group, followed by the S group’s participants, while the highest percentage decrease in lean mass was noted in the C group’s participants [22].

**Figure 1:**
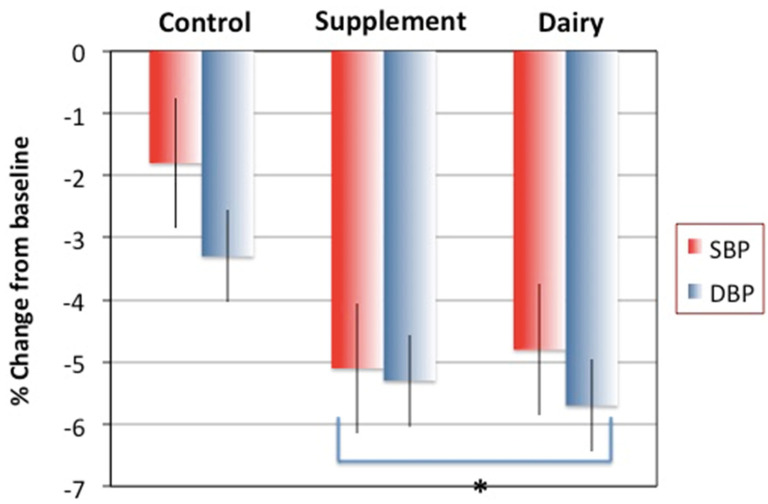
Percent change (Mean − SEM) in systolic and diastolic blood pressure (SBP and DBP), respectively. All *p* ≤ 0.05 compared to baseline (paired *t*-tests; for intragroup). Asterisk indicates significant difference for Supplement and Dairy groups’ participants vs. Control (*p* ≤ 0.05 by ANOVA followed by Bonfferoni corrections; for intergroup) at 6 months. Control/placebo, *n* = 30; Supplement, *n* = 37; Dairy, *n* = 30.

Regarding the outcomes of the secondary analyses, Figure 1 presents the percent change in blood pressure after 6 months in each group. Although initial blood pressure was close to normal in all participants, it decreased significantly in each group after 6 months of intervention (*p* ≤ 0.05, intragroup). Moreover, the participants in the S and D groups ended with significantly lower values for both SBP and DBP at 6 months compared to the C group’s participants (*p* ≤ 0.05, intergroup).

Table 3 presents the baseline and 6-month values of relevant serum biomarkers in participants stratified by study groups with intra- (within the same group at baseline and/or 6 months) and inter- (within the different groups at baseline and/or 6 months) differences. There was no significant difference in glucose among the groups at baseline and it did not change over time, while insulin decreased in C and S groups’ participants after 6 months. Among other variables at baseline, only leptin was significantly higher in C group’s participants and remained such. After 6 months, leptin decreased, and adiponectin increased significantly in all groups (both expected with weight loss). ApoA1 increased and ApoB decreased significantly in all groups, while hs-CRP remained the same. Serum 25(OH)D significantly increased in S and D groups (also expected, although not necessarily reflected in the dietary records for D group). All other clinical parameters used as health indicators (electrolytes, minerals, not presented) were within the normal ranges and remained such after 6 months.

**Figure 2:**
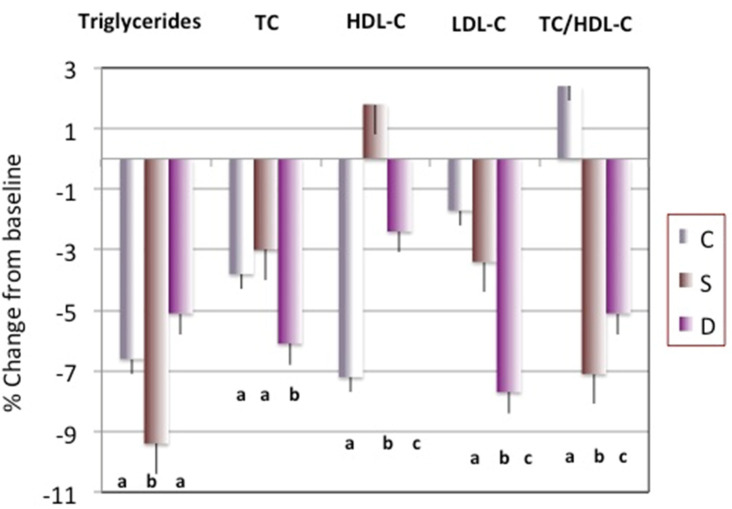
Percent change (Mean − SEM) in triglycerides, total cholesterol (TC), high density lipoproteins cholesterol (HDL-C) low density lipoproteins cholesterol (LDL-C), and ratio of TC/HDL-C. Different letters indicate significant difference among groups (*p* ≤ 0.05 by ANOVA followed by Bonfferoni corrections). C, Control/placebo, *n* = 30; S, Supplement, *n* = 37; D, Dairy, *n* = 30.

Figure 2 presents percentage change in serum lipid variables after 6 months. Overall, serum lipids improved in all groups, presumably due to the loss of weight/fat. However, the reduction in triglycerides was the highest in the S group, the reduction in total and LDL-C was the highest in the D group, while the increase in the HDL-C was the highest in the S group’s participants and so was the decrease in the TC/HDL-C ratio (all *p* ≤ 0.05).

Detailed dietary and physical activity variables at baseline and 6 months in the participants stratified by the study groups have been published in the original study ([22], in Table 3). The same trends were noted in this smaller fraction of the population. Most importantly, after 6 months, energy and macronutrient intake significantly decreased compared to baseline, while Ca intake significantly decreased in C, but increased in S and D groups’ participants, compared to baseline. Vitamin D intake was unchanged in C and D groups, while it significantly increased in the S group’s participants compared to baseline. Of note is that the D group’s participants consumed about 2/3 of their total dairy foods as yogurts and cheese (fermented) and about 1/3 as milk (non-fermented). In the overall population, ~30% of participants did not drink milk at all, or not in any considerable amount.

## 4. Discussion

Most of the examined cardiometabolic indices improved in all participants, including those in the control (C) group, due to the weight loss. A significant improvement in C group’s participants after 6 months was noted in BP, triglycerides, TC, insulin, leptin, adiponectin, ApoA1, ApoB and their ratio (Figure 1 and Figure 2, and Table 3). However, our results reflect significantly better outcomes in some indices in participants belonging to both Ca + vitamin D supplements (S) and low-fat dairy (D) groups. Despite the significant decrease in both SBP and DBP in all participants, the decrease was significantly greater in the S and D, compared to the C group’s participants (Figure 1). The highest percentage decrease in triglycerides and TC/HDL-C ratio was noted in the S group, while the highest percentage decrease in TC and LDL-C was in the D group’s participants. The percentage of HDL-C increased only in the S group, while the percentage of TC/HDL-C ratio increased in the C group’s participants (Figure 2).

Blood pressure can often be manipulated with lifestyle changes [33], including weight loss, an overall healthy dietary pattern, and physical activity. Some of these changes could have been the case for all participants in our study. Although they were on average normotensive, a significant decrease in blood pressure, particularly DBP, was impressive. The results from an earlier meta-analysis of 40 studies showed that Ca supplements of ~1 g/day reduced both SBP and DBP and the effect was even larger in those with lower initial Ca intake [34]. Similarly, the results from the most recent Cochrane review of some 18 clinical trials and 3140 normotensive participants showed the dose response: higher Ca intake (1–1.5 g/day) resulted in a higher reduction in SBP [21]. The results of our study are in agreement with the results of both reviews. On the contrary, the results from the secondary analysis of the Women Health Initiative, Ca/vitamin D supplements arm, showed no effect of the supplements on blood pressure compared to placebo in postmenopausal women during a 7-year follow-up [35]. Such findings could be attributed to the number of limitation (pointed by the authors), of which probably the most important were non-adherence during a long-time follow-up and some participants taking open label supplements, thus muddying the results.

Although dairy foods are more complex in their action and their concomitant health effects (see more below), a recent study showed the clear benefits of their intake on blood pressure. Overweight men and women were enrolled in the cross-over intervention study with high or low dairy foods during a period of 6 weeks. The results showed that those consuming higher amounts of dairy foods experienced a significant decrease in both SBP and DBP, although the effect might have been dependent on the coexistent higher calcium intake [36]. The authors also speculate that, in addition to the synergistic action of other nutrients in dairy, one of the important and unique contributors in dairy foods could be the presence of the amino acid cysteine, producing hydrogen sulfide and eventually nitrogen monoxide, both potent vasodilators. Our results corroborate these findings, persisting even for the longer time (6 months), which is especially important in the current time of the COVID-19 pandemic. The results from a new study in over 460,000 men and women, participants of U.S. employer-sponsored wellness programs, revealed the significant increase in both SBP and DBP during the lockdown period (March–December 2020) compared to 2019 (pre-pandemic time), particularly in women of 65–88 years of age [37]. In view of this, awareness of some relatively simple dietary approaches to reduce blood pressure is important to avoid further pandemic and/or post-pandemic complications in general population.

In addition to weight loss in its own right, both calcium and dairy food consumption have been implicated in BP lowering effects. The calcium effect could be explained by the common link between calciotropic hormones, namely, PTH and calcitriol (1,25(OH)_2_ vitamin D), and BP regulators, namely, renin-angiotensin-aldosterone system (RAAS). Each of these pathways is regulated by intracellular calcium, which depends on serum (ionized extracellular) calcium that is ultimately influenced by calcium intake [27,38]. Briefly, with low calcium intake, serum calcium decreases promoting the synthesis of 1,25(OH)_2_ vitamin D and the secretion of PTH, the latter activating RAAS. A rise in serum 1,25(OH)_2_ vitamin D and PTH promotes a higher influx of calcium inside the cells causing its increased concentration. This is known as “calcium paradox” (higher intracellular calcium is caused by lower serum calcium/intake) [39]. Simultaneously, the RAAS activated by the rise in PTH promotes vasoconstriction, leading to peripheral vascular resistance and increased BP. A similar mechanism with low serum calcium (due to low intake) promoting a higher influx of intracellular calcium (via PTH and 1,25(OH)_2_ vitamin D) into adipocytes has been attributed to decreased lipolysis and weight gain, explaining how higher calcium intake may promote weight maintenance or weight loss [40].

Regarding the changes in serum lipids (triglycerides and cholesterol fractions), our results point to better outcomes in the S and D groups’ participants (Figure 2). Other, but not all, studies showed similar improvements with either calcium supplements or dairy foods. As discussed above, increased calcium intake (with or without energy restriction) may help in lipid metabolism by inhibiting lipogenesis in adipocytes and promoting lipolysis, decreasing circulatory free fatty acids and promoting their oxidation [40]. In addition, although probably with a lower magnitude, calcium effect could be linked to the absorptive pathways in which dietary calcium binds fatty acids in the intestinal tract and limits their absorption [41].

As far as dairy foods modifying circulating lipids are concerned, the EPIC–Norfolk study reported changes in dairy consumption parallel with changes in cardiometabolic markers in over 15,600 adults (40–78 years old), following them from baseline to an average of 3.7 years [7]. Among other beneficial outcomes associated with higher intake of variety of dairy foods (high fat, low fat, and milk) consumption, the authors reported higher HDL-C and lower LDL-C in those with higher intakes [7]. Not all studies are consistent and there are also some null or negative findings [5]. However, corroborating our findings, the results from the recent review of several studies [42] revealed that calcium intake, either as a supplement or part of dairy matrix, is in most cases associated with lower TC and LDL-C, and emphasized that the mechanisms of actions are multifaceted, operating within the pathways of fat absorption and digestion, as well as interacting with bile salts and their subsequent excretion.

Other notable findings of our study include the significant decrease in serum ApoB and leptin and increase in ApoA1 and adiponectin in all groups (Table 3). Among apoproteins, ApoB and ApoA1 are the primary protein components of non-HDL-C (LDL-C, IDL-C, VLDL-C) and HDL-C, respectively. Elevated ApoB and lowered ApoA1 are associated with an increased risk of CVDs and may be even better indicators of lipid abnormalities than their corresponding lipoprotein-cholesterol fractions [43,44]. An earlier large meta-analysis showed that the ApoB/ApoA1 ratio (presenting the balance between atherogenic and anti-atherogenic apoproteins) was an even better risk indicator than the traditional serum lipids [45]. Similarly, a study in normotensive, healthy, young-to-middle age men showed that the ApoB/ApoA1 ratio was more discriminatory in detecting atherogenic lipid profile [46]. In our study, participants in all groups ended up with significantly lower ApoB and higher ApoA1, leading to an improved ApoB/ApoA1 ratio, all resulting in normal values for each after 6 months (Table 3).

Both leptin and adiponectin are adipokines/hormones released primarily from adipose tissue, but the former is also produced in vascular smooth muscle cells and its concentration is elevated both in obesity and in CVDs, including hypertension. Conversely, adiponectin is a cardioprotective hormone and has been shown to reduce oxidative stress and inflammation. When within normal serum concentrations, leptin may act in reducing body weight by regulating appetite and increasing energy expenditure [47]. However, in hyperleptinemia and subsequent development of leptin resistance, it fails to modulate appetite and exerts pro-inflammatory properties and, as such, is implicated not just in propagating obesity [48], but also in atherogenesis and arterial stiffness, thus increasing the risks for CVDs development [49]. All our participants had elevated serum leptin concentration (due to being overweight/obese); it significantly decreased in all groups after 6 months, but never reached normal/recommended levels. It was recently hypothesized that dysregulated leptin levels may compromise the immune system in COVID-19 patients and worsen the outcomes of the infection [50]. Thus, the trend in our study was beneficial for our participants, but also points to the need for health professionals to monitor serum leptin in relation to excess adiposity, particularly during the pandemic.

In contrast to leptin and other adipokines, adiponectin is beneficial for cardiometabolic diseases and its serum concentrations and is inversely associated with adiposity and directly with insulin sensitivity, rendering its normal levels crucial for optimal physiological actions in the cardiovascular system [47]. The participants of our study, although having normal serum adiponectin concentrations at baseline, experienced a significant increase after the intervention (no difference among the groups), which positioned them at a more favorable health status.

In our study participants, glucose (normal at baseline) and hs-CRP (elevated at baseline) did not change over time, while insulin significantly decreased in the C and S groups, but not in the D group’s participants (Table 3). It is well established that CRP concentrations increase in response to tissue injury or inflammation and hs-CRP has been endorsed by multiple guidelines as a biomarker of atherosclerotic/cardiovascular disease risk [43,51]. Some clinical trials demonstrated a significantly lower cardiovascular risk for patients with hs-CRP lower than 2.0 mg/L [43]. These guidelines implicate that our participants (with serum hs-CRP concentrations above the acceptable normal values) might have been at elevated inflammatory state, which did not change with weight loss or intervention.

Circulating serum 25(OH)D increased in all participants of our study and significantly so in S and D groups (Table 3), as would be expected due to the intervention. The increase in the C group’s participants (and to a certain extent in others) is most likely due to its release from the fat stores during the weight/body fat loss. Although the changes were small, and participants had normal values to begin with, they may indicate an improvement in some CVDs risk indices, as well as good compliance with the prescribed diets. Ample evidence suggests a critical role of low serum 25(OH)D in tissue inflammation and numerous cardiometabolic diseases risks, including atherosclerotic plaque formation, metabolic syndrome, and diabetes, as well as an increased risk of myocardial infarction and stroke in postmenopausal women [52,53]. Such observations are supported by some biologically plausible mechanisms, including vitamin D receptors that are found on endothelial and smooth muscle cells, showing that circulating 25(OH)D improved endothelial function by reducing the production of proinflammatory cytokines, as well as lowering PTH levels and RAAS activity [54]. Therefore, the positive outcomes of our intervention on serum 25(OH)D are encouraging.

It is interesting to note that, after 6 months, serum PTH slightly increased in the C, but decreased in the S and D groups’ participants, becoming significantly lower in the S group compared to baseline, again as expected due to calcium supplementation. Other studies have also shown a reduction in serum PTH and increases in 25(OH)D upon calcium and vitamin D supplementation [55,56], although the data are not consistent for dairy foods in young women [57]. As often reported, maintaining lower PTH improves other CVDs risks outcomes and PTH in older individuals has been posited as vasculo-toxic in its own right [58,59]. Since all participants in our study had normal baseline levels of serum PTH, the clinical importance of this change might not be relevant, other than possibly for BP, as discussed above.

Regarding the mechanism of dairy foods mediating weight/adiposity and subsequent cardiometabolic risk factors, in addition to calcium, several other dairy components have been considered, all possibly acting synergistically. Although sometimes controversial, they include whey peptides [60], conjugated linoleic acid [61], and branched chain amino acids [62]. Other contributing factors include the regulation of appetite via calcium and its absorption of fatty acids [41], or improvement of metabolic activity in gut microbiota [31]. Some health benefits have been attributed to milk itself, including those for type 2 diabetes [63], colorectal and prostate cancers, and blood pressure [64]. Conversely, other studies showed higher mortality in both men and women, as well higher incidence of fractures in women [8] or higher incidence of prediabetes and diabetes [65] with higher milk consumption. Some of these unfavorable effects associated with high milk consumption could be attributed to its galactose content (a proinflammatory monosaccharide [9]). However, most of these studies were observational cohort studies and should be interpreted with caution. Overall, there is abundant evidence (corroborated also with our findings) that points to the collective beneficial roles of dairy foods acting on multiple levels and improving the outcomes of several chronic diseases. Some of these include cardiometabolic diseases (e.g., reducing blood pressure, decreasing diabetes and metabolic syndrome risks [63,66,67]) and obesity (e.g., by inhibiting lipogenesis [40]).

There are several limitations, as well as strengths, to our study. There was a considerable attrition rate as well as missing data points. It is well established that the problems with adherence to the study protocol and fidelity are inherent in any human study with intervention and follow-up. Multiple measures were employed to counteract the above mentioned adversities and were described in the original study [22]. It was recently reported [68] that the manipulation of missing data could be problematic. Therefore, in order to obtain more straight-forward results, in this secondary analysis, we used only complete data sets without substituting for missing data, which could have decreased the power even more. However, the interventional study with yogurt (used for our power estimation [31]) resulted in significant positive outcomes in serum lipids with *n* = 30 participants/group. Our study was conducted in Caucasian overweight/obese early postmenopausal women (as per inclusion criteria), which limits the generalizability, but increases the homogeneity of the study, particularly regarding the cardiometabolic risk factors that are different among different ethnic and sex groups (e.g., Caucasian vs. African Americans or women vs. men). The actual/objective measurements (bone and body composition, blood and urine sample analyses) and extensive data collection on other lifestyle factors (including dietary and physical activity assessments) enabled the comprehensive analyses and inclusion of multiple confounders.

## 5. Conclusions

The results from this secondary analysis show that hypocaloric diets with Ca + vitamin D supplements (S) and/or low-fat dairy foods (D) during the 6-month intervention resulted in better outcomes of some cardiometabolic risk factors compared to only hypocaloric diets in control (C) group, although the latter group experienced some favorable changes as well. This is in view of the participants’ moderate weight and body fat loss, as detailed earlier [22], and lower than what was reported in some other studies with a similar design [69].

Despite some controversies concerning the role of dairy foods, including milk and calcium/vitamin D supplements in some health issues, our study supports the notion that the adequate intake of either dairy foods or calcium/vitamin D, as complements to hypocaloric diets may protect against or improve cardiometabolic diseases risks in overweight/obese postmenopausal women.

## Figures and Tables

**Table 1:** Baseline characteristics, body composition, serum analyzes, dietary intake of selected nutrients and physical activity (Mean ± SD) of participants who finished the 6 month follow-up with complete data sets.

Variables	*n* = 97	
Age (y)	55.8 ± 3.7	
Years Since Menopause (y)	6.6 ± 3.4	
Blood Pressure		Reference
Systolic blood pressure (mmHg)	121.5 ± 15.1	<120
Diastolic blood pressure (mmHg)	80.0 ± 9.4	<80
Anthropometry/Body Composition		
Weight (kg)	83.0 ± 13.5	
Height (cm)	163.5 ± 5.8	
Body Mass Index (kg/m^2^)	31.0 ± 4.8	
Total body fat (kg) Total body fat (%) Total body lean mass (kg)	37.8 ± 10.5 45.4 ± 4.8 42.1 ± 5.1	
Serum Parameters		Reference Range
Glucose (mg/dL)	100.6 ± 17.3	65–99
Insulin (µIU/mL)	10.0 ± 8.8	<17
Triglycerides (mg/dL)	135.8 ± 75.8	<149
TC (mg/dL)	214.2 ± 42.4	100–199
LDL-C (mg/dL)	130.3 ± 39.9	<99
HDL-C (mg/dL)	57.0 ± 17.6	>39
TC/HDL-C ratio	4.1 ± 1.4	3.5–5
Leptin (ng/mL)	38.9 ± 26.0	3–18
Adiponectin (µg/mL)	12.1 ± 6.0	5–30
ApoA1 (mg/dL)	85.4 ± 69.3	100–205
ApoB (mg/dL)	114.2 ± 49.1	<100
ApoB/ApoA1	1.3 ± 0.7	0.8–0.9
hs-CRP (mg/L)	4.7 ± 5.0	1–3
25(OH)D (nmol/L)	65.6 ± 28.0	50–125
Parathyroid hormone (pmol/L)	3.1 ± 1.6	1.6–6.9
Energy and Selected Nutrients		DRI
Energy (kcal/day)	1769.2 ± 426.1	
Protein (g/day)	73.9 ± 20.0	
Protein (g/kg/day)	0.90 ± 1.6	0.8
Carbohydrates (g/day)	209.0 ± 60.3	
Fat (g/day)	68.7 ± 23.2	
Total calcium (mg/day) *	905.6 ± 383.9	1200
Total vitamin D (IU/day) *	343.6 ± 288.2	600–800
Physical Activity		
Total activity (h/week) **	7.9 ± 6.8	
MET	44.1 ± 5.3	

* Includes the amount from food and from the multivitamin/mineral supplements taken at baseline. ** Includes housework, gardening, do-it-yourself activities, walking and recreation. TC, total cholesterol; LDL-C, Low density lipoproteins cholesterol; HDL-C, High density lipoproteins cholesterol; ApoA1, Apoproteins A1; ApoB, Apoproteins B; hs-CRP, High sensitivity C-reactive protein; 25(OH)D, 25 hydroxy vitamin D; DRI, Dietary Reference Intakes; MET, Metabolic equivalent.

**Table 2:** Baseline Pearson correlation coefficients (r) and *p*-values, between body composition and serum parameters in participants who finished the 6-month intervention.

Serum Parameters	Weight		BMI		Body Fat (kg)		Body Fat (%)		Lean Mass (kg)	
	r	*p*	r	*p*	r	*p*	r	*p*	r	*p*
SBP	0.111	ns	0.234	0.0196	0.144	ns	0.138	ns	0.031	ns
DBP	0.093	ns	0.204	0.0431	0.101	ns	0.117	ns	0.032	ns
Glucose	0.223	0.0258	0.211	0.0352	0.190	ns	0.135	ns	0.247	0.0131
Insulin	0.422	≤0.0001	0.474	≤0.0001	0.393	≤0.0001	0.233	0.0211	0.402	≤0.0001
Triglycerides	0.139	ns	0.223	0.0258	0.118	ns	0.079	ns	0.148	ns
TC	−0.009	ns	−0.024	ns	−0.044	ns	−0.026	ns	0.007	ns
LDL-C	0.039	ns	−0.005	ns	0.035	ns	0.037	ns	0.035	ns
HDL-C	−0.189	ns	−0.223	0.0258	−0.189	ns	−0.175	ns	−0.167	ns
TC/HDL-C	0.183	ns	0.185	ns	0.130	ns	0.105	ns	0.203	0.0430
Leptin	0.534	≤0.0001	0.604	≤0.0001	0.593	≤0.0001	0.581	≤0.0001	0.296	0.0028
Adiponectin	0.035	ns	−0.033	ns	0.015	ns	−0.051	ns	0.039	ns
ApoA1	−0.158	ns	−0.110	ns	−0.127	ns	−0.070	ns	−0.182	ns
ApoB	0.220	0.0289	0.254	0.0111	0.203	0.0453	0.122	ns	0.220	0.0289
hs-CRP	0.438	≤0.0001	0.467	≤0.0001	0.493	≤0.0001	0.431	≤0.0001	0.274	0.0057
25(OH)D	−0.204	0.0386	−0.181	ns	−0.212	0.0351	−0.229	0.0216	−0.110	ns
PTH	0.035	ns	0.067	ns	0.066	ns	0.156	ns	−0.071	ns

BMI, Body mass index; SBP, Systolic blood pressure; DBP, Diastolic blood pressure; TC, total cholesterol; LDL-C, Low density lipoproteins cholesterol; HDL-C, High density lipoproteins cholesterol; ApoA1, Apoproteins A1; ApoB, Apoproteins B; hs-CRP, High sensitivity C-reactive protein; 25(OH)D, 25 hydroxy vitamin D; PTH, parathyroid hormone.

**Table 3:** Serum biomarkers in participants divided in intervention groups at baseline and after 6 months (mean ± SD); *n* = 30, *n* = 37, *n* = 30 for control/placebo (C), supplement (S) and dairy (D) group, respectively.

Variables ^†^	Baseline			*p* ^ab^	6 Months			*p* ”
	C	S	D		C	S	D	
Glucose (mg/dL)	104.4 ± 16.9	97.3 ± 7.2	98.5 ± 8.6	NS	101.1 ± 11.2	95.7 ± 8.3	97.2 ± 10.4	NS
Insulin (µIU/mL)	11.2 ± 10.9	9.6 ± 8.9	9.2 ± 6.7	NS	9.7 ” ± 5.8	8.3 ”^a^ ± 5.4	9.3 ± 7.1	
Leptin (ng/mL)	46.4 ^a^ ± 34.9	32.5 ^b^ ± 19.1	37.8 ^b^ ± 19.1		37.9 ”^a^ ± 26.3	28.9 ”^b^ ± 16.9	29.5 ”^b^ ± 21.7	
Adiponectin (µg/mL)	11.7 ± 5.2	11.8 ± 5.9	12.7 ± 6.5	NS	17.9 ” ± 8.5	16.4 ” ± 9.3	15.4 ” ± 5.8	
ApoA1 (mg/dL)	77.4 ± 55.3	86.9 ± 65.3	84.1 ± 63.5	NS	102.8 ”^a^ ± 59.9	117.8 ”^b^ ± 78.7	105.6 ”^a^ ± 60.0	
ApoB (mg/dL)	121.4 ± 56.5	114.0 ± 43.7	117.6 ± 42.4	NS	95.6 ” ± 36.5	97.8 ” ± 31.8	92.9 ” ± 27.5	
ApoB/ApoA1	1.6 ± 1.0	1.3 ± 0.7	1.4 ± 0.7		0.9 ” ± 0.6	0.8 ” ± 0.4	0.9 ” ± 0.5	
hs-CRP (mg/L)	4.8 ± 4.7	4.6 ± 5.7	4.8 ± 4.7	NS	4.8 ± 5.3	4.6 ± 5.7	4.9 ± 6.5	NS
Serum 25(OH)D (nmol/L)	65.8 ± 24.4	70.7 ± 32.7	66.6 ± 27.5	NS	71.9 ^a^ ± 30.9	83.0 ”^b^ ± 23.4	82.8 ”^b^ ± 46.8	
Parathyroid hormone (pmol/L)	3.0 ± 1.4	3.1 ± 1.7	3.2 ± 1.8	NS	3.5 ± 1.3	2.8 ” ± 1.2	3.0 ± 0.9	

^†^ Values are adjusted for age and body mass index (BMI); ^†^ By ANOVA and subsequent *t*-tests; ApoA1, apolipoprotein A1; ApoB, apolipoprotein B, hs-CRP, high sensitivity C-reactive protein; 25(OH)D, 25 hydroxy vitamin D. *p* ^ab^ Statistically significant difference among the groups (intergroup) at baseline and/or 6 months (2 sample *t*-test). *p* ” Statistically significant difference within the group (intragroup) at 6 months compared to the baseline (paired *t*-test).

## References

[1] WHO CVD Risk Chart Working Group (2019). World Health Organization Cardiovascular Disease Risk Charts: Revised Models to Estimate Risk in 21 Global Regions. Lancet Glob. Health.

[2] JafariNasabian P., Inglis J.E., Reilly W., Kelly O.J., Ilich J.Z. (2017). Aging Human Body: Changes in Bone, Muscle and Body Fat with Consequent Changes in Nutrient Intake. J. Endocrinol..

[3] Furman D., Campisi J., Verdin E., Carrera-Bastos P., Targ S., Franceschi C., Ferrucci L., Gilroy D.W., Fasano A., Miller G.W. (2019). Chronic inflammation in the etiology of disease across the life span. Nat. Med..

[4] U.S. Department of Health and Human Services, U.S. Department of Agriculture (2015). 2015–2020 Dietary Guidelines for Americans.

[5] Guo J., Astrup A., Lovegrove J.A., Gijsbers L., Givens D.I., Soedamah-Muthu S.S. (2017). Milk and Dairy Consumption and Risk of Cardiovascular Diseases and All-Cause Mortality: Dose-Response Meta-Analysis of Prospective Cohort Studies. Eur. J. Epidemiol..

[6] Sonestedt E., Wirfält E., Wallström P., Gullberg B., Orho-Melander M., Hedblad B. (2011). Dairy Products and Its Association with Incidence of Cardiovascular Disease: The Malmö Diet and Cancer Cohort. Eur. J. Epidemiol..

[7] Trichia E., Luben R., Khaw K.-T., Wareham N.J., Imamura F., Forouhi N.G. (2020). The Associations of Longitudinal Changes in Consumption of Total and Types of Dairy Products and Markers of Metabolic Risk and Adiposity: Findings from the European Investigation into Cancer and Nutrition (EPIC)-Norfolk Study, United Kingdom. Am. J. Clin. Nutr..

[8] Michaëlsson K., Wolk A., Langenskiöld S., Basu S., Lemming E.W., Melhus H., Byberg L. (2014). Milk Intake and Risk of Mortality and Fractures in Women and Men: Cohort Studies. BMJ.

[9] Cui X., Wang L., Zuo P., Han Z., Fang Z., Li W., Liu J. (2004). D-Galactose-Caused Life Shortening in Drosophila Melanogaster and Musca Domestica Is Associated with Oxidative Stress. Biogerontology.

[10] Astrup A. (2008). The Role of Calcium in Energy Balance and Obesity: The Search for Mechanisms. Am. J. Clin. Nutr..

[11] Bolland M.J., Leung W., Tai V., Bastin S., Gamble G.D., Grey A., Reid I.R. (2015). Calcium Intake and Risk of Fracture: Systematic Review. BMJ.

[12] Bolland M.J., Grey A., Avenell A., Gamble G.D., Reid I.R. (2011). Calcium Supplements with or without Vitamin D and Risk of Cardiovascular Events: Reanalysis of the Women’s Health Initiative Limited Access Dataset and Meta-Analysis. BMJ.

[13] Cannata-Andía J.B., Carrillo-López N., Messina O.D., Hamdy N.A.T., Panizo S., Ferrari S.L., on behalf of the International Osteoporosis Foundation (IOF) Working Group on Bone and Cardiovascular Diseases (2021). Pathophysiology of Vascular Calcification and Bone Loss: Linked Disorders of Ageing?. Nutrients.

[14] Lewis J.R., Calver J., Zhu K., Flicker L., Prince R.L. (2011). Calcium Supplementation and the Risks of Atherosclerotic Vascular Disease in Older Women: Results of a 5-Year RCT and a 4.5-Year Follow-Up. J. Bone Miner. Res..

[15] Lewis J.R., Radavelli-Bagatini S., Rejnmark L., Chen J.S., Simpson J.M., Lappe J.M., Mosekilde L., Prentice R.L., Prince R.L. (2015). The Effects of Calcium Supplementation on Verified Coronary Heart Disease Hospitalization and Death in Postmenopausal Women: A Collaborative Meta-Analysis of Randomized Controlled Trials. J. Bone Miner. Res..

[16] Kim K.J., Kim M.S., Hong N., Bae J.H., Kim K.J., Kim N.H., Rhee Y., Lee J., Kim S.G. (2021). Cardiovascular Risks Associated with Calcium Supplementation in Patients with Osteoporosis: A Nationwide Cohort Study. Eur. Heart J. Cardiovasc. Pharm..

[17] Gallo L., Faniello M.C., Canino G., Tripolino C., Gnasso A., Cuda G., Costanzo F.S., Irace C. (2016). Serum Calcium Increase Correlates With Worsening of Lipid Profile: An Observational Study on a Large Cohort From South Italy. Medicine.

[18] Derakhshandeh-Rishehri S.-M., Ghobadi S., Akhlaghi M., Faghih S. (2020). The Effect of Calcium Supplement Intake on Lipid Profile: A Systematic Review and Meta-Analysis of Randomized Controlled Clinical Trials. Crit. Rev. Food Sci. Nutr..

[19] Cormick G., Belizán J.M. (2019). Calcium Intake and Health. Nutrients.

[20] Gil Á., Ortega R.M. (2019). Introduction and Executive Summary of the Supplement, Role of Milk and Dairy Products in Health and Prevention of Noncommunicable Chronic Diseases: A Series of Systematic Reviews. Adv. Nutr..

[21] Cormick G., Ciapponi A., Cafferata M.L., Cormick M.S., Belizán J.M. (2021). Calcium Supplementation for Prevention of Primary Hypertension. Cochrane Database Syst. Rev..

[22] Ilich J.Z., Kelly O.J., Liu P.-Y., Shin H., Kim Y., Chi Y., Wickrama K.K.A.S., Colic-Baric I. (2019). Role of Calcium and Low-Fat Dairy Foods in Weight-Loss Outcomes Revisited: Results from the Randomized Trial of Effects on Bone and Body Composition in Overweight/Obese Postmenopausal Women. Nutrients.

[23] Mifflin M.D., St Jeor S.T., Hill L.A., Scott B.J., Daugherty S.A., Koh Y.O. (1990). A New Predictive Equation for Resting Energy Expenditure in Healthy Individuals. Am. J. Clin. Nutr..

[24] Shin H., Shin J., Liu P.-Y., Dutton G.R., Abood D.A., Ilich J.Z. (2011). Self-Efficacy Improves Weight Loss in Overweight/Obese Postmenopausal Women during a 6-Month Weight Loss Intervention. Nutr. Res..

[25] Perri M.G., Nezu A.M., McKelvey W.F., Shermer R.L., Renjilian D.A., Viegener B.J. (2001). Relapse Prevention Training and Problem-Solving Therapy in the Long-Term Management of Obesity. J. Consult. Clin. Psychol..

[26] Ilich J.Z., Blanusa M., Orlić Z.C., Orct T., Kostial K. (2009). Comparison of Calcium, Magnesium, Sodium, Potassium, Zinc, and Creatinine Concentration in 24-h and Spot Urine Samples in Women. Clin. Chem. Lab. Med..

[27] Lemacks J.L., Ilich J.Z., Liu P.-Y., Shin H., Ralston P.A., Cui M., Wickrama K.A.S. (2016). Dietary Influence on Calcitropic Hormones and Adiposity in Caucasian and African American Postmenopausal Women Assessed by Structural Equation Modeling (SEM). J. Nutr. Health Aging.

[28] Ilich J.Z., Brownbill R.A. (2008). Habitual and Low-Impact Activities Are Associated with Better Bone Outcomes and Lower Body Fat in Older Women. Calcif. Tissue Int..

[29] Bouchard C., Tremblay A., Leblanc C., Lortie G., Savard R., Thériault G. (1983). A Method to Assess Energy Expenditure in Children and Adults. Am. J. Clin. Nutr..

[30] Chao D., Espeland M.A., Farmer D., Register T.C., Lenchik L., Applegate W.B., Ettinger W.H. (2000). Effect of Voluntary Weight Loss on Bone Mineral Density in Older Overweight Women. J. Am. Geriatr. Soc..

[31] Ejtahed H.S., Mohtadi-Nia J., Homayouni-Rad A., Niafar M., Asghari-Jafarabadi M., Mofid V., Akbarian-Moghari A. (2011). Effect of Probiotic Yogurt Containing Lactobacillus Acidophilus and Bifidobacterium Lactis on Lipid Profile in Individuals with Type 2 Diabetes Mellitus. J. Dairy Sci..

[32] Faul F., Erdfelder E., Buchner A., Lang A.-G. (2009). Statistical Power Analyses Using G*Power 3.1: Tests for Correlation and Regression Analyses. Behav. Res. Methods.

[33] Cvijetic S., Kern J., Vuletic S., Ilich J.Z. (2020). Lifestyle Characteristics Influencing Hypertension in Middle-Age to Old People: Comparison of Two Populations. Arter. Hypertens..

[34] van Mierlo L.A., Arends L.R., Streppel M.T., Zeegers M.P.A., Kok F.J., Grobbee D.E., Geleijnse J.M. (2006). Blood Pressure Response to Calcium Supplementation: A Meta-Analysis of Randomized Controlled Trials. J. Hum. Hypertens..

[35] Margolis K.L., Ray R.M., Van Horn L., Manson J.E., Allison M.A., Black H.R., Beresford S.A.A., Connelly S.A., Curb J.D., Grimm R.H. (2008). Effect of Calcium and Vitamin D Supplementation on Blood Pressure: The Women’s Health Initiative Randomized Trial. Hypertension.

[36] Rietsema S., Eelderink C., Joustra M.L., van Vliet I.M.Y., van Londen M., Corpeleijn E., Singh-Povel C.M., Geurts J.M.W., Kootstra-Ros J.E., Westerhuis R. (2019). Effect of High Compared with Low Dairy Intake on Blood Pressure in Overweight Middle-Aged Adults: Results of a Randomized Crossover Intervention Study. Am. J. Clin. Nutr..

[37] Laffin L.J., Kaufman H.W., Chen Z., Niles J.K., Arellano A.R., Bare L.A., Hazen S.L. (2021). Rise in Blood Pressure Observed Among US Adults During the COVID-19 Pandemic. Circulation.

[38] Villa-Etchegoyen C., Lombarte M., Matamoros N., Belizán J.M., Cormick G. (2019). Mechanisms Involved in the Relationship between Low Calcium Intake and High Blood Pressure. Nutrients.

[39] Fujita T., Palmieri G.M. (2000). Calcium paradox disease: Calcium deficiency prompting secondary hyperparathyroidism and cellular calcium overload. J. Bone Miner. Metab..

[40] Zemel M.B., Shi H., Greer B., Dirienzo D., Zemel P.C. (2000). Regulation of adiposity by dietary calcium. FASEB J..

[41] Scharger S. (2005). Dietary Calcium Intake and Obesity. J. Am. Board Fam. Pract..

[42] Mulet-Cabero A.-I., Wilde P.J. (2021). Role of Calcium on Lipid Digestion and Serum Lipids: A Review. Crit. Rev. Food Sci. Nutr..

[43] Reiner Z., Catapano A.L., De Backer G., Graham I., Taskinen M.-R., Wiklund O., Agewall S., Alegria E., Chapman M.J., European Association for Cardiovascular Prevention & Rehabilitation (2011). ESC/EAS Guidelines for the Management of Dyslipidaemias: The Task Force for the Management of Dyslipidaemias of the European Society of Cardiology (ESC) and the European Atherosclerosis Society (EAS). Eur. Heart J..

[44] McQueen M.J., Hawken S., Wang X., Ounpuu S., Sniderman A., Probstfield J., Steyn K., Sanderson J.E., Hasani M., Volkova E. (2008). Lipid.ds, Lipoproteins, and Apolipoproteins as Risk Markers of Myocardial Infarction in 52 Countries (the INTERHEART Study): A Case-Control Study. Lancet.

[45] Thompson A., Danesh J. (2006). Associations between Apolipoprotein B, Apolipoprotein AI, the Apolipoprotein B/AI Ratio and Coronary Heart Disease: A Literature-Based Meta-Analysis of Prospective Studies. J. Intern. Med..

[46] Kaneva A.M., Potolitsyna N.N., Bojko E.R., Odland J.Ø. (2015). The Apolipoprotein B/Apolipoprotein A-I Ratio as a Potential Marker of Plasma Atherogenicity. Dis. Markers.

[47] Ghantous C.M., Azrak Z., Hanache S., Abou-Kheir W., Zeidan A. (2015). Differential Role of Leptin and Adiponectin in Cardiovascular System. Int. J. Endocrinol..

[48] Iikuni N., Lam Q.L.K., Lu L., Matarese G., La Cava A. (2008). Leptin and Inflammation. Curr. Immunol. Rev..

[49] Katsiki N., Mikhailidis D.P., Banach M. (2018). Leptin, Cardiovascular Diseases and Type 2 Diabetes Mellitus. Acta Pharmacol. Sin..

[50] Rebello C.J., Kirwan J.P., Greenway F.L. (2020). Obesity, the Most Common Comorbidity in SARS-CoV-2: Is Leptin the Link?. Int. J. Obes..

[51] Goff D.C., Lloyd-Jones D.M., Bennett G., Coady S., D’Agostino R.B., Gibbons R., Greenland P., Lackland D.T., Levy D., O’Donnell C.J. (2014). 2013 ACC/AHA Guideline on the Assessment of Cardiovascular Risk: A Report of the American College of Cardiology/American Heart Association Task Force on Practice Guidelines. Circulation.

[52] Norman P.E., Powell J.T. (2014). Vitamin D and Cardiovascular Disease. Circ. Res..

[53] Kendrick J., Targher G., Smits G., Chonchol M. (2009). 25-Hydroxyvitamin D Deficiency Is Independently Associated with Cardiovascular Disease in the Third National Health and Nutrition Examination Survey. Atherosclerosis.

[54] Beveridge L.A., Struthers A.D., Khan F., Jorde R., Scragg R., Macdonald H.M., Alvarez J.A., Boxer R.S., Dalbeni A., Gepner A.D. (2015). Effect of Vitamin D Supplementation on Blood Pressure: A Systematic Review and Meta-analysis Incorporating Individual Patient Data. JAMA Intern. Med..

[55] McCarty M.F., Thomas C.A. (2003). PTH Excess May Promote Weight Gain by Impeding Catecholamine-Induced Lipolysis-Implications for the Impact of Calcium, Vitamin D, and Alcohol on Body Weight. Med. Hypotheses.

[56] Pfeifer M., Begerow B., Minne H.W., Nachtigall D., Hansen C. (2001). Effects of a Short-Term Vitamin D_3_ and Calcium Supplementation on Blood Pressure and Parathyroid Hormone Levels in Elderly Women. J. Clin. Endocrinol. Metab..

[57] Gunther C.W., Legowski P.A., Lyle R.M., McCabe G.P., Eagan M.S., Peacock M., Teegarden D. (2005). Dairy Products Do Not Lead to Alterations in Body Weight or Fat Mass in Young Women in a 1-y Intervention. Am. J. Clin. Nutr..

[58] Kestenbaum B., Katz R., de Boer I., Hoofnagle A., Sarnak M.J., Shlipak M.G., Jenny N.S., Siscovick D.S. (2011). Vitamin D, Parathyroid Hormone, and Cardiovascular Events among Older Adults. J. Am. Coll. Cardiol..

[59] Hagström E., Ingelsson E., Sundström J., Hellman P., Larsson T., Berglund L., Melhus H., Held C., Michaëlsson K., Lind L. (2010). Plasma Parathyroid Hormone and Risk of Congestive Heart Failure in the Community. Eur. J. Heart Fail..

[60] Frestedt J.L., Zenk J.L., Kuskowski M.A., Ward L.S., Bastian E.D. (2008). A Whey-Protein Supplement Increases Fat Loss and Spares Lean Muscle in Obese Subjects: A Randomized Human Clinical Study. Nutr. Metab..

[61] Eder K., Ringseis R. (2010). Metabolism and Actions of Conjugated Linoleic Acids on Atherosclerosis-related Events in Vascular Endothelial Cells and Smooth Muscle Cells. Mol. Nutr. Food Res..

[62] Layman D.K. (2003). The Role of Leucine in Weight Loss Diets and Glucose Homeostasis. J. Nutr..

[63] Liu S., Choi H.K., Ford E., Song Y., Klevak A., Buring J.E., Manson J.E. (2006). A Prospective Study of Dairy Intake and the Risk of Type 2 Diabetes in Women. Diabetes Care.

[64] Yu E., Hu F.B. (2018). Dairy Products, Dairy Fatty Acids, and the Prevention of Cardiometabolic Disease: A Review of Recent Evidence. Curr Atheroscler. Rep..

[65] Brouwer-Brolsma E.M., Sluik D., Singh-Povel C.M., Feskens E.J.M. (2018). Dairy Product Consumption Is Associated with Pre-Diabetes and Newly Diagnosed Type 2 Diabetes in the Lifelines Cohort Study. Br. J. Nutr..

[66] Azadbakht L., Mirmiran P., Esmaillzadeh A., Azizi F. (2005). Dairy Consumption Is Inversely Associated with the Prevalence of the Metabolic Syndrome in Tehranian Adults. Am. J. Clin. Nutr..

[67] Faghih S., Abadi A.R., Hedayati M., Kimiagar S.M. (2011). Comparison of the Effects of Cows’ Milk, Fortified Soy Milk, and Calcium Supplement on Weight and Fat Loss in Premenopausal Overweight and Obese Women. Nutr. Metab. Cardiovasc. Dis..

[68] Li P., Stuart E.A. (2019). Best (but Oft-Forgotten) Practices: Missing Data Methods in Randomized Controlled Nutrition Trials. Am. J. Clin. Nutr..

[69] Zemel M.B., Thompson W., Milstead A., Morris K., Campbell P. (2004). Calcium and Dairy Acceleration of Weight and Fat Loss during Energy Restriction in Obese Adults. Obes. Res..

